# Characterizing the effect of temperature fluctuation on the incidence of malaria: an epidemiological study in south-west China using the varying coefficient distributed lag non-linear model

**DOI:** 10.1186/1475-2875-13-192

**Published:** 2014-05-27

**Authors:** Xing Zhao, Fei Chen, Zijian Feng, Xiaosong Li, Xiao-Hua Zhou

**Affiliations:** 1 West China School of Public Health, Sichuan University, No.17 Section 3, South Renmin Road, 610041 Chengdu, China; 2 Department of Biostatistics, School of Public Health, University of Washington, NE Pacific Street, Seattle 98195, USA; 3 Office for Disease Control and Emergency Response, Chinese Centre for Disease Control and Prevention, NE Pacific Street, 102206 Beijing, China; 4 HSR&D Center of Excellence, VA Puget Sound Health Care System, 1100 Olive Way Metro Park West Suite 1400, Seattle 98101, USA

## Abstract

**Background:**

Malaria transmission is strongly determined by the environmental temperature and the environment is rarely constant. Therefore, mosquitoes and parasites are not only exposed to the mean temperature, but also to daily temperature variation. Recently, both theoretical and laboratory work has shown, in addition to mean temperatures, daily fluctuations in temperature can affect essential mosquito and parasite traits that determine malaria transmission intensity. However, so far there is no epidemiological evidence at the population level to this problem.

**Methods:**

Thirty counties in southwest China were selected, and corresponding weekly malaria cases and weekly meteorological variables were collected from 2004 to 2009. Particularly, maximum, mean and minimum temperatures were collected. The daily temperature fluctuation was measured by the diurnal temperature range (DTR), the difference between the maximum and minimum temperature. The distributed lag non-linear model (MDLNM) was used to study the correlation between weekly malaria incidences and weekly mean temperatures, and the correlation pattern was allowed to vary over different levels of daily temperature fluctuations.

**Results:**

The overall non-linear patterns for mean temperatures are distinct across different levels of DTR. When under cooler temperature conditions, the larger mean temperature effect on malaria incidences is found in the groups of higher DTR, suggesting that large daily temperature fluctuations act to speed up the malaria incidence in cooler environmental conditions. In contrast, high daily fluctuations under warmer conditions will lead to slow down the mean temperature effect. Furthermore, in the group of highest DTR, 24-25°C or 21-23°C are detected as the optimal temperature for the malaria transmission.

**Conclusion:**

The environment is rarely constant, and the result highlights the need to consider temperature fluctuations as well as mean temperatures, when trying to understand or predict malaria transmission. This work may be the first epidemiological study confirming that the effect of the mean temperature depends on temperature fluctuations, resulting in relevant evidence at the population level.

## Background

Climate plays a crucial role in the dynamics and distribution of malaria [1,2]. From the biological perspective, climate is intrinsically linked to malaria incidence through its effects on both the mosquito vector and the development of the malaria parasite inside the mosquito vector [3]. Although rainfall and relative humidity can help provide fitting habitats for mosquitoes to breed, temperature can determine not only the mosquito's development and biting rate, but also the development speed and survival of the parasites within the mosquito [4].

Currently, most epidemiological research studying the relationship between temperature and malaria is based solely on mean temperatures, such as mean monthly temperatures. However, recent theoretical work [5-7] and laboratory empirical study [8] demonstrated that in addition to the mean temperature, the temperature variation that occurs throughout the day also affects several aspects of the transmission. The laboratory empirical study [8] shows that daily temperature fluctuations influence the parasite infection, the rate of parasite development, mosquito biology, and ultimately determine the transmission process. Similar results were also found for dengue [9,10]. These studies demonstrated that daily temperature fluctuation around the cooler temperatures acts to speed up the rate process, whereas the fluctuation around high mean temperatures acts to slow down processes. However, all above results were derived either through theoretical thermodynamic models using only temperature data, or from laboratory empirical study. There is no epidemiological study at the population level to examine whether the association between the mean temperatures and malaria incidence does depend on the daily temperature fluctuation. It is worth investigating this problem at the population level.

When modelling the climatic effect on malaria cases, special attention is required for two problems, non-linear and lagged patterns. On the one hand, the single fixed lag assumption was not plausible for describing population level associations [11,12]. Biologically speaking, there are several periods to be considered for the lag effect, such as the time for mosquitoes to develop, the development period of parasites within the mosquito, and the incubation period of parasites within human body. Climatic factors will influence most of the stages. For example, higher temperatures may reduce the time for larval development, and larvae may react with different intensities to temperatures. Consequently, the association between climatic factors and malaria cases shall show a variation in terms of the time lag, resulting in a smoothly varied lag distribution at population level. On the other hand, the non-linear effect was recognized in temperatures, and substantial existing studies validated the non-linear correlation between temperatures and malaria in terms of laboratory and epidemiological studies [2,13-16]. Similar potential non-linear correlations were also proposed to rainfall [15,17,18]. As a result, those two patterns should be taken into account in the regression model. Distributed lag non-linear model provides a useful approach for this problem [19].

The goal of the current paper is using both temperature and malaria incidence data to study whether the association between the mean temperatures and malaria incidence does depend on the diurnal temperature range (DTR). No epidemiological study regarding this problem was reported before. Specifically, a distributed lag non-linear model (DLNM) was used to study the correlation between weekly mean temperatures and weekly malaria incidences using data from 2004 to 2009 in 30 counties in southwest China. In addition, the correlation pattern was allowed to vary depending on the weekly mean DTR. The result can help understanding of the association between temperatures and malaria transmission, testing the biological hypothesis in terms of epidemiological level.

## Methods

### Study sites

Malaria remains a significant public health issue in the southern part of mainland China. Particularly, Yunnan Province used to be the highest endemic province [20]. For southwest China, the majority of previous studies focused on spatiotemporal pattern for mortality or morbidity [21-23], or pathogenic classifications of reported cases [24]. Southwest China (21°14′to 34°31′N, 97°35′to 110°19′E) consists of four provinces, Sichuan, Chongqing, Yunnan, and Guizhou. The area has a population of 189,977,077 (sixth national census in 2010) and encompasses 1,137,570 sq km. There are 483 counties (county-level cities and districts). Thirty counties were selected as the study sites based on availability of malaria and meteorological data. The malaria data covered the 483 counties while only 131 counties had the daily meteorological record; a detailed description of these datasets is in the next section. The set of counties with both malaria and meteorological data were sorted by the average annual incidences, and the top 30 counties were included in the analysis. See Additional file 1 for a map of the 483 counties in southwest China and the selected 30 counties.

### Data description

Meteorological data were collected from the publicly available Chinese Meteorological Data Sharing Service System [25]. This system was constructed by Chinese National Meteorological Information Centre. There are 836 meteorological monitoring stations with the daily record in the whole of China, 131 in the southwest. Roughly speaking, three to four counties (438/131) share one monitoring station to monitor the daily meteorological information, and no county has two monitoring stations. No information indicates that some stations have better data than others, and they are national level stations, hence they should have similar data qualities. The monitoring station should suffice to represent where the county is, an assumption usually made in existing studies [16,22,26-29]. Those monitoring stations located in counties with malaria prevalence and corresponding counties were used.

Five kinds of daily meteorological data, from July 2003 to December 2009, were obtained for the 30 selected counties. They are maximum, mean and minimum temperature, rainfall, and relative humidity. Temperatures and rainfall variables are in °C and mm, respectively. The daily temperature fluctuation is measured by the DTR, which was calculated as the difference between the maximum and minimum temperature on each day. Weekly mean values were calculated by averaging the corresponding daily values over each week. The proportion of missing meteorological data is very low. The highest missing proportion occurred to the maximum temperature with missing rate less than 0.001. The missing data were imputed by the mean value of the two closest non-missing values from the same monitoring station.

Weekly malaria cases in the 30 counties were obtained from 2004 to 2009 from the Chinese Centre for Disease Control and Prevention (CCDC). At county level, it is not unreasonable to assume that malaria heterogeneity is not great, a usual assumption from existing studies [23,30,31]. Moreover, since the interest is on the effect of temperature variables, the heterogeneity caused by other factors should not affect the result, unless other factors are related to the temperature variable. The malaria data collection were facilitated by the Chinese Information System for Infectious Diseases Control and Prevention (CISIDCP). CISIDCP was established on the basis of individual cases and public health emergencies. A virtual private network (VPN) was constructed, and information on individual cases is directly reported to the national database through the internet. This system covers all health data sources and reports new malaria cases to CCDC within 24 hours [32]. Although malaria cases observed in the 30 counties included *Plasmodium vivax* and *Plasmodium falciparum*, most data did not separate different parasites. Population data for every county from 2004 to 2009 were retrieved from the National Bureau of Statistics of China.

### Basic distributed lag non-linear model

DLNM represent a modelling framework to describe simultaneously non-linear and delayed dependencies [19]. As mentioned in Background, the motivation for the lag effect is the realization that temperatures can affect not merely cases occurring on one week, but on several subsequent weeks. Therefore, the converse is also true: cases of this week will depend on temperatures of many weeks before, and the final contribution of temperatures is the cumulative effect of preceding weeks. Similar interpretations also apply to other climatic variables. As with ordinary count data, Poisson regression was used to model the association between the expected number of cases *E*(*Y*_*it*_) in week t in county *i* and the meteorological factors in the previous weeks,

(1) $$\begin{array}{ll} \log(E\left(Y_{\mathrm{it}}\right))= & \log(d_{\mathrm{it}})+\beta_{i0}\\ & +\sum_{l=3}^{10}f\left(x_{i\left(t-l\right),T_{m}},\beta_{T_{m}l}\right)\\ & +\sum_{l=4}^{15}f\left(x_{i\left(t-l\right),r},\beta_{\mathrm{rl}}\right)\\ & +\sum_{l=4}^{15}f\left(x_{i\left(t-l\right),h},\beta_{\mathrm{hl}}\right), \end{array}$$

Here, *d*_*it*_ is the population in county *i* in week *t*; *β*_*i*0_ is the intercept effect for county *i*. The climatic variables for county *i* in week *t* are $x_{\mathrm{it},T_{m}}$, *x*_*it*,*r*_ and *x*_*it*,*h*_, denoting the weekly mean temperature, the weekly rainfall and the weekly mean relative humidity, respectively.

Biological considerations suggest the lag ranges for meteorological factors. As mentioned, Model (1) accounts for the cumulative contributions from the time interval specified by the lag range, instead of assuming a single fixed lag time. Those lag ranges were chosen mainly according to [11], which gave both the biological reasoning and the empirical study. For example, at 16°C the larval development may take 47 days, and the sporogonic cycle may take 111 days. Besides, there are ten to 16 days for the incubation period in human. See [11] for the full reasoning. Eventually, three to ten weeks were used for the weekly mean temperature, while four to 15 weeks were used for the weekly rainfall and the weekly mean relative humidity [11,18,33].

In addition to the lag ranges, Model (1) involves two basis functions for the non-linear and lag effects, respectively. Take the mean temperature for example, one function is $f\left(x_{i\left(t-l\right),T_{m}},\beta_{T_{m}l}\right)$, which is the non-linear effect of the mean temperature *l* weeks before. Many functional forms can be chosen for *f*(*x*_*i*(*t* − *l*),*r*_, *β*_*rl*_), such as the polynomial function. The other function is to constrain the parameter $\beta_{T_{m}l}$. Since there is a substantial correlation between mean temperatures on weeks close together, the above regression will have a high degree of collinearity, which will result in unstable estimates of the individual βTml's. To gain more efficiency and more insight into the distributed effect of mean temperature over time, it is useful to constrain the βTml's. If this is done flexibly, substantial gains in reducing the noise of the unconstrained distributed lag model can be obtained, with minimal bias [12].

The second-order natural cubic spline was used for both the non-linear and lag effects of meteorological variables. This choice was partly due to the prior knowledge of the unimodal pattern for meteorological variables [15,17,34], and partly due to the requirement of parsimony.

Finally, correlations within one county would be greater over those between counties due to some unmeasured (or perhaps unmeasurable) county-specific covariates, and therefore *β*_*i*0_ took a multilevel structure random intercept, which was a normal distribution with a mean of *β*_0_ and a variance of $\sigma_{0}^{2}$. *β*_0_ is the average intercept over all counties, and $\sigma_{0}^{2}$ characterizes the variation of county-specific intercepts around the average intercept.

$$\beta_{i0}\sim N\left(\beta_{0},\sigma_{0}^{2}\right),$$

In a previous study [35], instead of mean temperatures, maximum and minimum temperatures were included in Model (1) to examine the lagged pattern between malaria cases and meteorological factors.

### Varying coefficient distributed lag non-linear model

The temperature fluctuation was not included in Model (1), or implicitly, the model assumes the mean temperature has the same effect over different level of temperature fluctuations. However, the pattern of mean temperatures may depend on the temperature fluctuation. To relax this assumption, a varying coefficient model [36] was applied to examine whether the effect of mean temperature depends on the temperature fluctuation.

The functional form for the lag pattern is over the entire three to ten weeks for the mean temperature, indicating that any variation during three to ten weeks before would influence the whole functional form. Let *x*_*it*,*f*_ denote the average DTR over the three to ten weeks before week *t*, and therefore *x*_*it*,*f*_ should, to some extent, determine the lag non-linear pattern, provided the pattern of mean temperature does depend on the temperature fluctuation.

To examine the possible DTR influence, first all *x*_*it*,*f*_ were approximately equally divided into four quantile groups, using their 25, 50 and 75% percentiles. The four groups were defined as groups 0–3 respectively, and following dummy variables were created to indicate the group membership,

$$T_{\mathrm{it},\mathrm{fg}}=\left\{\begin{array}{l} 1\;\text{if}\;x_{\mathrm{it},f}\;\text{is}\;\mathrm{in}\;\mathrm{level}\;g\\ 0\;\text{otherwise} \end{array}\right)$$

Specifically, *T*_*it*,*f*0_ = 1 represents the *x*_*it*,*f*_ is at the first DTR level, also the lowest level, and *T*_*it*,*f*1_ = 1 represents the *x*_*it*,*f*_ is at the second DTR level. Similar interpretations apply to *T*_*it*,*f*2_ = 1 and *T*_*it*,*f*3_ = 1. To investigate the mean temperature pattern over different DTR levels, Model (1) is modified as follows:

(2) $$\begin{array}{ll} \log(E\left(Y_{\mathrm{it}}\right))= & \log(d_{\mathrm{it}})+\beta_{i0}+\sum_{g=1}^{3}\alpha_{g}\times T_{\mathrm{it},\mathrm{fg}}\\ & +\sum_{l=3}^{10}f\left(x_{i\left(t-l\right),T_{m}},\beta_{T_{m}l}\left(T_{\mathrm{it},\mathrm{fg}}\right)\right)\\ & +\sum_{l=4}^{15}f\left(x_{i\left(t-l\right),r},\beta_{\mathrm{rl}}\right)\\ & +\sum_{l=4}^{15}f\left(x_{i\left(t-l\right),h},\beta_{\mathrm{hl}}\right), \end{array}$$

There are two differences between Model (2) and Model (1). The major difference is that $\beta_{T_{m}l}$ is replaced by $\beta_{T_{m}l}\left(T_{\mathrm{it},\mathrm{fg}}\right)$, indicating the model coefficients $\beta_{T_{m}l}$ is varying over different level of DTR, *T*_*it*,*fg*_. As a result, the effect of mean temperatures depends on the corresponding level of temperature fluctuations. In addition to allowing for the lag non-linear effect of mean temperatures, this model can reveal the effect change over temperature fluctuations. Therefore, there should be four distinctive lag non-linear patterns for mean temperatures provided that the variation does exist. The second difference is the inclusion of *T*_*it*,*fg*_. Like the ordinary categorical predictor, the lowest level for *T*_*it*,*fg*_ was chosen as the reference group, with the remaining three parameters representing the difference effects with respect to the reference group. Specifically, *T*_*it*,*f*0_ was specified as the reference group, and *α*_1_, *α*_2_ and *α*_3_ represent difference effects for *T*_*it*,*f*1_, *T*_*it*,*f*2_ and *T*_*it*,*f*3_, respectively.

On the other hand, Model (2) made the same assumption as Model (1) for the rainfall and mean relative humidity. These two variables do not have interaction with the temperature fluctuation. Zero was used for all climatic factors as the reference value to report the result.

The result might be sensitive to the lag range specification. As sensitivity for the lag range, instead of the tenth week, the 12th week was also specified as the maximum lag range for temperatures.

All the implementations above were accomplished by R. R is a free software programming language and a software environment for statistical computing and graphics [37]. Specifically, the add-on packages lme4 [38] was used for the parameter estimation.

## Results

### Descriptive analysis

**Figure 1:**
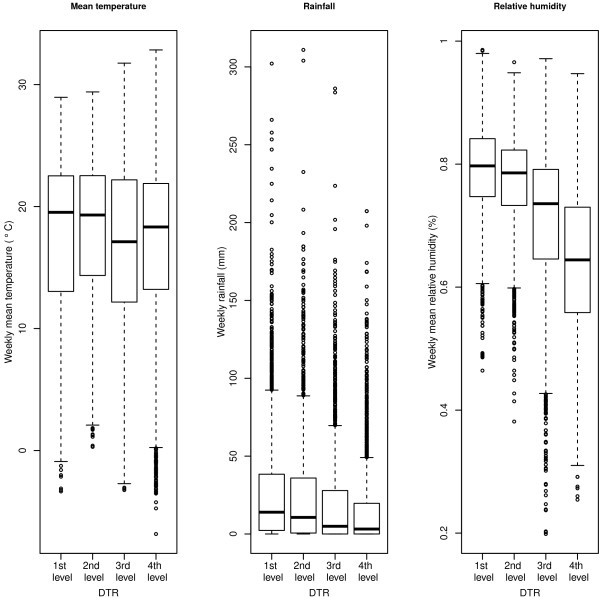
**Box plot comparison of meteorological variables between four diurnal temperature range levels.** The dark line in the middle of the boxes is the median value; the bottom and top of the boxes indicates the 25th and 75th percentile, respectively; whiskers represents 1.5 times the height of the box; and dots with numbers represent value of outlier cases.

From 2004 to 2009, 21,944 malaria cases were reported in the selected 30 counties in southwest China. Table 1 presents the descriptive analysis for the 30 counties.The four intervals defining the four levels of DTR are (3.93°C, 9.53°C), (9.53°C, 11.14°C), (11.14°C, 14.01°C), (14.01°C, 23.73°C), and their sample sizes are 2,351, 2,348, 2,344, and 2,347, respectively. Figure 1 demonstrates the comparison of meteorological variables between different levels of DTR. For the mean temperature, the median values of the two lower DTR groups are a little higher than those of the other two groups, and the overall difference is not large. On the contrary, there are pronounced trends for the rainfall and relative humidity among the four groups. They both show a decreasing pattern with the increase of DTR.

**Table 1:** Characteristics of the 30 study counties

**County**	**Cases**	**Annualized average incidences (/100,000)**	**Mean temperature☆ (°C)**	**DTR☆ (°C)**	**Rain☆☆ (mm)**	**Relative humidity☆ (%)**
Ruili	3,442	348.204	21.2 (17.7, 24.6)	10.8 (7.7,14)	26.53 (0, 43.03)	73.0 (67, 80)
Tengchong	9,255	246.049	20.4 (16, 24.5)	10.7 (7.8, 13.8)	17.80 (1, 24.7)	65.0 (54, 77)
Gongshan	300	136.897	6.7 (1.4,12.3)	10.8 (8, 13.8)	12.29 (2, 17.63)	69.6 (61, 80)
Fugong	455	80.657	12.3 (7.1, 17.7)	12.4 (9, 15.9)	16.59 (2, 27.85)	67.6 (58, 78)
Mengla	1,203	79.980	22.0 (18.9, 25.1)	11.3 (8.2,14.1)	28.02 (0, 41.83)	80.6 (77, 85)
Cangyuan	859	65.931	19.8 (16, 23.4)	12.3 (8.5, 16.3)	23.63 (0, 37.93)	72.4 (65, 81)
Menglian	735	55.274	20 (16.6, 23.1)	12.2 (8.4, 16.1)	32.56 (0, 51.6)	75.3 (70, 82)
Jinping	966	47.375	16.5 (12.7, 20.7)	7.3 (4.6, 9.6)	28.95 (2.25, 40.35)	84.8 (81, 91)
Longyang	1,976	37.041	16.6 (12.1, 20.7)	11.2 (7.9, 14.6)	17.94 (0.08, 27.23)	73.1 (66, 81)
Congjiang	688	34.928	19 (12.3, 25.7)	8.7 (5.1, 11.9)	22.03 (0.7, 33.8)	78.6 (73, 84)
Jiangcheng	142	22.283	19.1 (15.7, 22.4)	9.9 (6.7, 13)	41.89 (0.38, 69.28)	79.2 (76, 84)
Menghai	420	21.036	22.8 (19.8, 25.6)	11.8 (8.6, 14.5)	23.17 (0, 38.63)	77.4 (72, 84)
Weixi	174	18.725	7.0 (1.6, 12.8)	12.5 (7.9, 16.7)	11.76 (0, 17.93)	65.9 (58, 74)
Shuangjiang	113	10.580	18.3 (14.6, 21.6)	11.3 (7.8, 14.9)	21.22 (0.08, 32.63)	67.6 (59, 77)
Simao	119	8.132	19.3 (16.2, 22.3)	10.2 (7.2, 13.1)	27.18 (0, 43.8)	75.9 (71, 83)
Mojiang	173	7.565	24.1 (20.1, 28.1)	11.6 (8.4, 14.3)	15.40 (0, 21.4)	66.6 (59, 75)
Jingdong	166	7.382	19 (14.6, 23.1)	12.4 (8.3, 16.6)	22.21 (0.38, 31.23)	74.7 (70, 82)
Dechang	86	7.335	17.6 (13.1, 22.2)	11.4 (8.5, 14.4)	18.39 (0, 28.1)	59.3 (50, 71)
Gejiu	156	5.535	19.5 (16.1, 23.2)	8.7 (6.7, 10.6)	15.90 (0, 21.18)	68.3 (63, 75)
Dushan	102	4.984	15.6 (9.7, 22)	7.3 (4.6, 9.5)	23.94 (1.5, 32.68)	79.4 (73, 88)
Changshun	53	3.598	16.4 (10.3, 22.6)	8.1 (5.1, 10.5)	22.30 (1.48, 29.1)	77.5 (72, 84)
Liping	75	2.522	16.3 (9.2, 23.8)	7.8 (4.3, 10.9)	23.68 (1.9, 33.33)	81.3 (74, 90)
Wenshan	64	2.325	16.5 (12.9, 20.6)	9.6 (6.8, 12.1)	17.64 (0.5, 26.03)	78.3 (74, 85)
Wangmo	29	1.609	20.0 (14.8, 25.6)	9.1 (5.9, 11.8)	22.43 (0.5, 26.43)	73.2 (67, 80)
Guangnan	74	1.575	17.5 (13.1, 22.4)	10.0 (6.5, 13)	16.98 (0.5, 23.8)	76.8 (72, 84)
Weishan	28	1.482	15.5 (11.5, 19.5)	10.6 (8.2, 13.1)	20.37 (0, 33.28)	66.2 (55, 78)
Nanhua	19	1.318	16.5 (12.3, 20.5)	10.4 (7.6, 13.1)	15.66 (0, 24.08)	68.2 (59, 80)
Weng’an	32	1.263	15.9 (9.1, 22.8)	7.3 (3.8, 10.1)	19.65 (2.6, 27.85)	77.1 (71, 85)
Eshan	11	1.156	16.4(12.3, 20.3)	10.8 (7.8, 13.8)	16.23 (0, 23.63)	72.6 (67, 81)
Huili	29	1.089	15.6 (10.7, 20.3)	11.9 (8.2, 15.9)	21.53 (0, 27.95)	68.0 (60, 77)

☆: weekly mean, and the two values in the parenthesis are 25% and 75% percentiles, respectively.

☆☆: weekly total, and the two values in the parenthesis are 25% and 75% percentiles, respectively.

The annualized average incidences are 2.88, 3.651, 5.283, and 5.651 per 100,000 for the first to fourth DTR level, respectively. Therefore, the incidence rate was higher with higher DTR.

### Varying coefficient distributed lag non-linear model

Table 2 gives the estimate of the main effect of DTR levels, representing the logarithm value of the relative risk ratio (logRR) compared to the reference group. While *α*_2_ is positive, *α*_1_ and *α*_3_ are negative, indicating the third group has the highest relative risk compared with the other three groups. Besides, the first group has the second highest relative risk, while the fourth group presents the lowest relative risk. However, the three parameters have confidence intervals containing zero, meaning their differences with respect to the reference group is not statistically significant.

**Table 2:** The estimate of the main effect of diurnal temperature range levels

**Parameters**	**Estimate**	**Standard error**	**95% confidence interval**	
*α*_1_	−0.127	0.419	−0.949	0.695
*α*_2_	0.489	0.297	−0.093	1.071
*α*_3_	−0.494	0.302	−1.086	0.099

**Figure 2:**
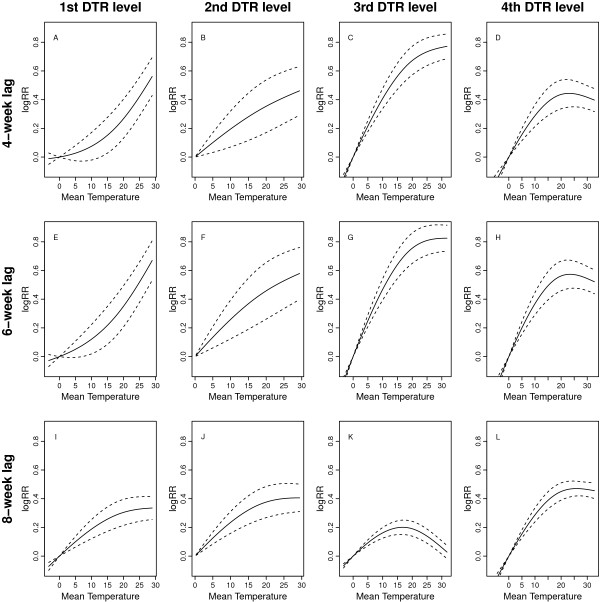
**The estimates of non-linear patterns between mean temperatures and malaria incidences, with three to ten weeks being the lag range of temperatures.** The Y-axis represents the logarithm value of the relative risk ratio compared to the reference temperature 0°C. The solid line is the estimated non-linear curve, with dashed lines indicating its 95% confidence interval. On the one hand, **A**, **B**, **C**, **D** show the scenario for the fourth week lag; **E**, **F**, **G**, **H** show the scenario for the sixth week lag; and **I**, **J**, **K**, **L** show the scenario for the eighth week lag. On the other hand, **A**, **E**, **I** are at the first (lowest) DTR level; **B**, **F**, **J** are at the second DTR level; **C**, **G**, **K** are at the third DTR level; and **D**, **H**, **L** are at the fourth (highest) DTR level. The range of X-axis depends on the corresponding range of mean temperatures.

Figure 2 shows the estimates of distributed lag non-linear relationships between mean temperatures and malaria incidences, and three to ten weeks was used as the lag range for mean temperature in Figure 2. Additional file 2 gives the same result while specifying three to12 weeks as the lag range. The results are presented in terms of the combination of three lags and four DTR levels. The Y-axis represents the logarithm value of the relative risk ratio compared to the reference temperature 0°C.

First, in Figure 2, for each time lag the four DTR levels present distinct non-linear patterns between mean temperatures and malaria incidences, and the highest DTR level shows an inverse-U shape. More specifically, in the highest DTR group when the mean temperature is greater than approximate 24°C, the logRR starts declining for all lags, and the inverse-U shape can also be observed at the eighth week lag in the second highest DTR group, while the coefficient levels off for the fourth and sixth weeks lags in the second highest DTR group. Those patterns are slightly marked in Additional file 2, in which the inverse-U shape is more evident in the highest DTR level. By contrast, in Figure 2 there is a non-decreasing pattern for mean temperatures in the two lower DTR groups. To be more specific, at the fourth and sixth weeks lags, in the two lower DTR groups, the logRR shows a steadily increasing tendency with the increase mean temperatures, while at the eighth week lag the logRR tends to increase at a lower rate when the mean temperature is relatively high. In Additional file 2, the logRR presents a constant increasing trend for all lags in the two lower DTR groups.

Second, from Figure 2 and Additional file 2, when less than 20°C, the mean temperature in the two higher DTR groups has a sharper ‘slope’ compared with those of two lower DTR groups, indicating a faster increasing trend in the higher daily fluctuation groups.

Third, in Figure 2, the cut-off value for the increasing to decreasing inverse ranges from 24°C to 25°C in the highest DTR group, while in Additional file 2 it ranges from 21°C to 23°C in the highest DTR group.

Fourth, in both Figure 2 and Additional file 2, the sixth week lag has the largest correlation within each of the four DTR levels, and the fourth week lag shows a larger correlation than the eighth week lag.

Contrasting Figure 2 and Additional file 2 shows that the general pattern is robust with respect to the lag range for the mean temperature.

## Discussion

Temperature is an important determinant for the dynamics and distribution of malaria. Despite extensive laboratory knowledge acquired on both the vector and the parasite, important questions remain on the precise role and interactions of the various biological processes driven by temperature [39-41].

Laboratory empirical studies have shown that the daily temperature fluctuation can affect both the mosquito [8,42] and parasites [8]. In particular, [8] provides empirical evidence that temperature fluctuations can affect all of the essential mosquito and parasite traits that determine malaria transmission intensity. Based on these laboratory studies, it is anticipated that similar results should be found in the epidemiological study.

The results find that the correlation between malaria incidence and mean temperature depends on daily temperature fluctuations. When under cooler temperature conditions, the larger mean temperature effect on malaria incidences is found in the groups of higher DTR, suggesting that a large daily temperature fluctuation acts to speed up the malaria incidence in cooler environmental conditions. On the other hand, under the warmer condition, high daily fluctuation will lead to slow down the mean temperature effect, which which can be observed in the highest DTR group and some lags in the second highest group in Figure 2 and Additional file 2. These results are consistent with the previous theoretical [5,6] and empirical laboratory studies [8]. In particular, the following explanation from the laboratory study [8] can be used to explain the pattern in this study. The key mosquito- and parasite-related traits determining malaria transmission intensity are all sensitive to daily variation in temperature, including parasite infection, parasite growth and development, immature mosquito development and survival, length of the gonotrophic cycle, and adult survival. In general, fluctuation increases relative rate processes under cool conditions and slows rate processes under warm conditions. The pattern in this work may result from the effect of one, or a combination of these mosquito- and parasite-related traits. However, as a limitation of the epidemiological study, this work cannot tell which traits have the effect.

The optimal mean temperature for the malaria transmission are detected as 24-25°C or 21-23°C, depending on the choices of lag ranges. For extrinsic incubation period (EIP), 21°C was identified by [5] as the point of inflection, and 25-28°C were identified as the optimal mean temperatures [5]. With the mean temperature of <21°C, temperature fluctuations could speed up the parasite development, whereas temperature fluctuations could slow the development with the mean temperature of >21°C. Moreover, 25°C was found by [4] to be to the optimal mean temperature for malaria transmission after considering several transmission parameters, such as the bite rate, the parasite development rate. The result agrees with those studies by finding similar optimal mean temperatures.

The lag has an effect increasing from the fourth to sixth weeks and decreasing from the sixth to eighth weeks, which is biologically plausible, as mean temperatures occurring on the same week or weeks too early before should not affect the malaria incidences in the current week.

*α*_1_, *α*_2_ and *α*_3_ are included for the main effect from the DTR level. It is noticeable from Figure 1 that the four groups of different DTR level do not have identical baseline distribution for the mean temperature, and therefore, the average effect should be distinct even under the same DTR condition. *α*_1_, *α*_2_ and *α*_3_ are used to represent this average difference, thus keeping all distributed lag non-linear curves starting from zero at the reference mean temperature. Consequently, the focus can be kept on the variation pattern for the mean temperature. Besides, the variation pattern of humidity and rainfall were not studied, as a previous study [35] has reported the results, and the scientific question here focuses on temperatures.

Instead of daily data, weekly data were used in this study for several reasons. First, the meteorological variables in close days should have similar values. Second, weekly malaria incidence can eliminate the possible week effect leading to the falsely elevated/reduced report rates in weekends. Third, daily data would give rise to a huge number of zero counts for cases compared to weekly data, which would make the parameter estimation unstable. Lastly, the lag range is well studied in the weekly scale.

Limitations of this study should be acknowledged. First, as with all observational studies for malaria and meteorological factors, it is likely that some confounding factors influence the result. Thirty counties might have different preventive measures (with different magnitudes) to combat malaria, and they may also have different behaviour habits, such as the use of nets. Including city-specific random effect could not eliminate the potential bias. Second, the quality and completeness of the data may change over the six-year period. The change mainly occurs in the time dimension [43-45], with the best quality in 2009 [45]. Third, the microclimate variation was not considered owing to the lack of relevant data. There are two kinds of variations, indoor air temperature *versus* outdoor air temperature [46], and water temperature *versus* air temperature [47]. However, it is not unreasonable to assume these factors do not present a systematic trend to confound the results in this study. Fourth, only the 30 counties with malaria prevalence were used in this study, and counties with zero malaria were not included in the analysis. However, this should not influence the result too much. It is evident from Table 1 that the 30 counties also included many low-incidence counties, such as Eshan with just 11 malaria cases for six years. The annualized average incidences range from 348.2/100,000 to 1.1/100,000. Fifth, the mosquito vector information did not exist in this study, hence this study cannot assess the impact. Finally, *P. vivax* and *P. falciparum* malaria could have different non-linear patterns. This study did not separate analyses by different parasites owing to a lack of detailed information on *P. vivax* and *P. falciparum* in this study. As pointed by [8], there is no reason to believe that the sensitivities of some parasites and mosquitoes are unique among malaria parasites and their mosquito vectors. Nonetheless, further epidemiological research is warranted to explore the possible different patterns.

## Conclusions

Using weekly malaria cases and meteorological information, this work studied the correlation between malaria incidences and mean temperature and temperature fluctuation over six years (2004–2009) in 30 counties in southwest China. This work may be the first epidemiological study confirming that the effect of mean temperatures depends on temperature fluctuation. Although as with other observational studies, the analysis cannot make direct cause-effect interpretation, the results can still be viewed as a supplementary evidence at the population level for the existing theoretic and laboratory evidence. The environment is rarely constant, and the result highlights the need to consider temperature fluctuations as well as mean temperatures, when trying to understand or predict malaria transmission.

## Competing interests

The authors declare that they have no competing interests.

## Authors’ contributions

XZ performed the statistical analysis and drafted the manuscript. XZ and FC cleared the data. ZJF provided the original data. XSL conceived of the project concept. XHZ gave technical assists. All of the authors have read and approved the final manuscript.

## Supplementary Material

Additional file 1 **Map of the 483 counties in southwest China and the selected 30 counties.** The grey-colored counties are the 30 top incidence counties with both malaria and meteorological data. Click here for file

Additional file 2 **The estimates of non-linear patterns between mean temperatures and malaria incidences, with three to 12 weeks being the lag range of temperatures.** The Y-axis represents the logarithm value of the relative risk ratio compared to the reference temperature 0°C. The solid line is the estimated non-linear curve, with dashed lines indicating its 95% confidence interval. On the one hand, **A**, **B**, **C**, **D** show the scenario for the fourth week lag; **E**, **F**, **G**, **H** show the scenario for the sixth week lag; and **I**, **J**, **K**, **L** show the scenario for the eighth week lag. On the other hand, **A**, **E**, **I** are at the first (lowest) DTR level; **B**, **F**, **J** are at the second DTR level; **C**, **G**, **K** are at the third DTR level; and **D**, **H**, **L** are at the fourth (highest) DTR level. The range of X-axis depends on the corresponding range of mean temperatures. Click here for file
