# Supplementary figures and images for: Characterizing the effect of temperature fluctuation on the incidence of malaria: an epidemiological study in south-west China using the varying coefficient distributed lag non-linear model
Source: Malar J. 2014 May 27;13:192. doi: 10.1186/1475-2875-13-192 (PMC4050477; doi:10.1186/1475-2875-13-192)

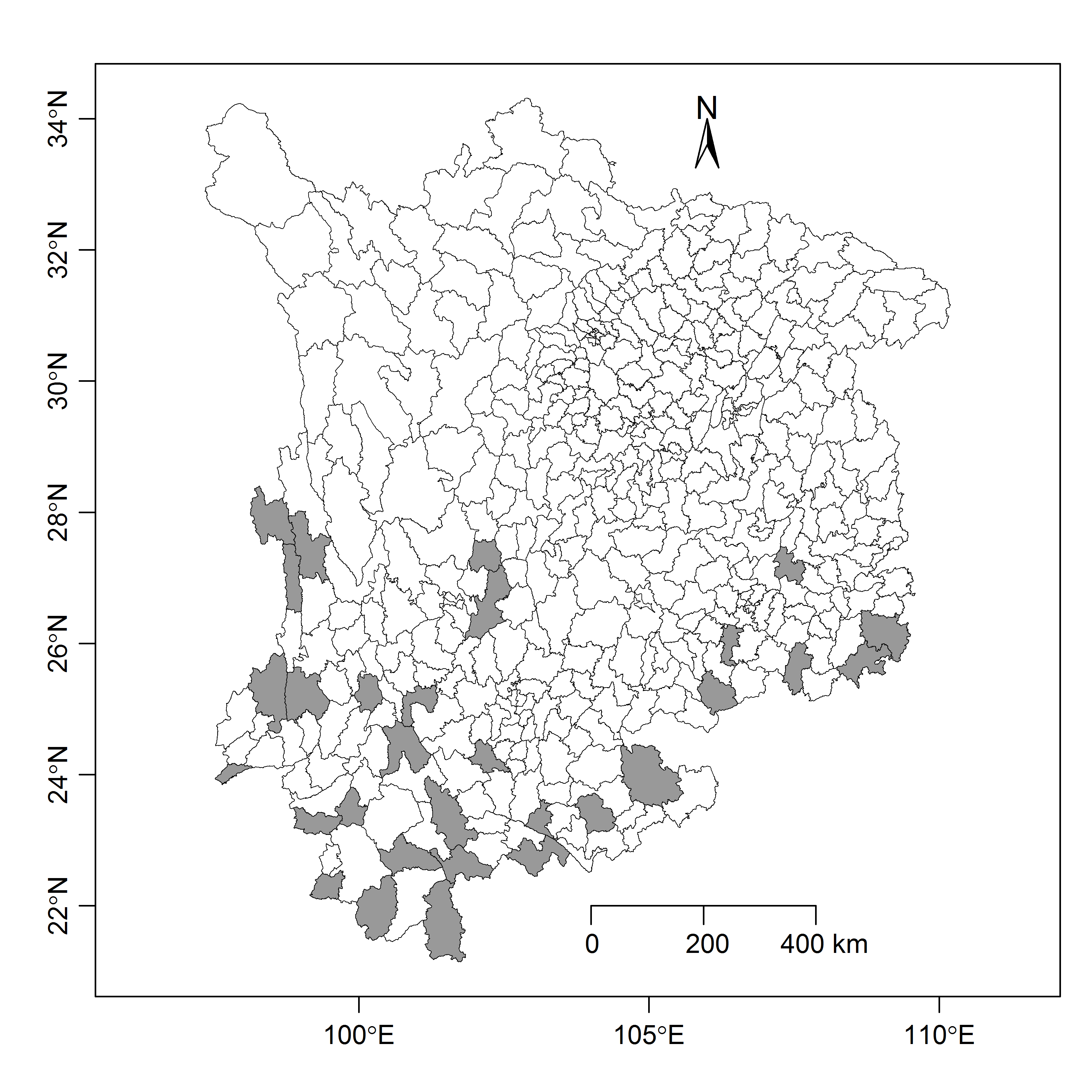

Supplement: Additional file 1 — Map of the 483 counties in southwest China and the selected 30 counties. The grey-colored counties are the 30 top incidence counties with both malaria and meteorological data. [file 1475-2875-13-192-S1.png]

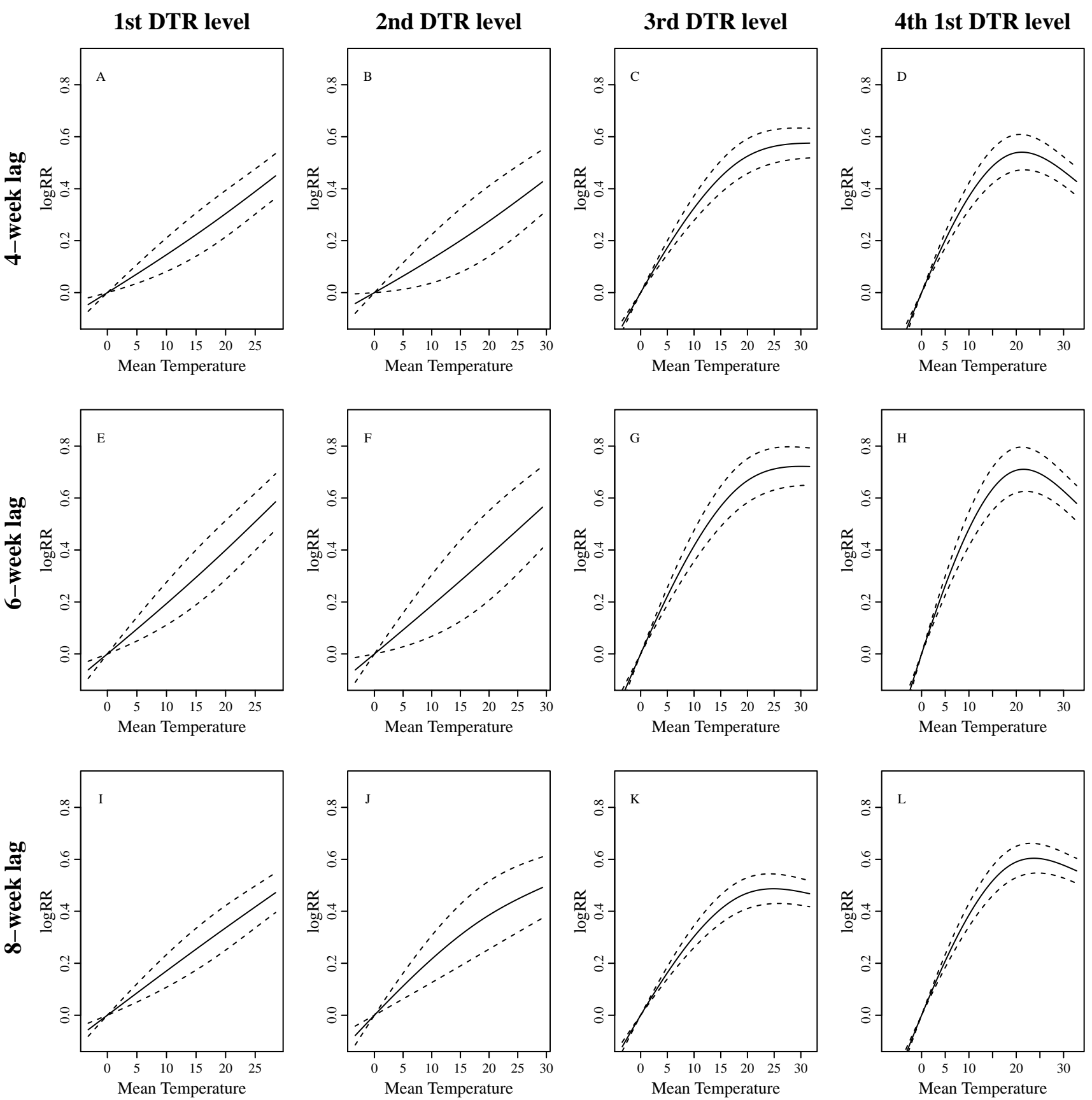

Supplement: Additional file 2 — The estimates of non-linear patterns between mean temperatures and malaria incidences, with three to 12 weeks being the lag range of temperatures. The Y-axis represents the logarithm value of the relative risk ratio compared to the reference temperature 0°C. The solid line is the estimated non-linear curve, with dashed lines indicating its 95% confidence interval. On the one hand, A, B, C, D show the scenario for the fourth week lag; E, F, G, H show the scenario for the sixth week lag; and I, J, K, L show the scenario for the eighth week lag. On the other hand, A, E, I are at the first (lowest) DTR level; B, F, J are at the second DTR level; C, G, K are at the third DTR level; and D, H, L are at the fourth (highest) DTR level. The range of X-axis depends on the corresponding range of mean temperatures. [file 1475-2875-13-192-S2.pdf]
